# Pacific Islands Families Study: Serum Uric Acid in Pacific Youth and the Associations with Free-Sugar Intake and Appendicular Skeletal Muscle Mass

**DOI:** 10.3390/nu17010054

**Published:** 2024-12-27

**Authors:** Shabnam Jalili-Moghaddam, Gael Mearns, Lindsay D. Plank, El-Shadan Tautolo, Elaine Rush

**Affiliations:** 1 National Institute for Stroke and Applied Neurosciences (NISAN), School of Clinical Sciences, Faculty of Health & Environmental Sciences, Auckland University of Technology, Auckland 0627, New Zealand; 2 School of Clinical Sciences, Auckland University of Technology, Auckland 1010, New Zealand; gael.mearns@aut.ac.nz; 3 Department of Surgery, Faculty of Medical and Health Sciences, University of Auckland, Auckland 1023, New Zealand; l.plank@auckland.ac.nz; 4 AUT Pacific Health Research Centre, School of Public Health and Interprofessional Studies, Auckland University of Technology, Auckland 1010, New Zealand; elshadan.tautolo@aut.ac.nz; 5 School of Sport and Recreation, Faculty of Health and Environmental Studies, Auckland University of Technology, Auckland 1010, New Zealand; elaine.rush@aut.ac.nz

**Keywords:** sugar intake, uric acid, body composition, adolescents, Pacific Islands

## Abstract

Background: Fructose (50% of sucrose/sugar) is one component of free-sugars and is metabolized to uric acid, which is a known risk factor for gout and metabolic syndrome. Pacific peoples in New Zealand experience a higher prevalence of gout, type 2 diabetes, and overweight/obesity than other ethnic groups. Interestingly, despite having a similar body mass index (BMI), they tend to have a higher proportion of appendicular skeletal muscle mass (ASMM) and less fat than other ethnic groups. Given this context, this study aimed to evaluate the associations between serum uric acid (SUA), free-sugar intake, and ASMM. Methods: In a nested sub-study from the Pacific Islands Families birth-cohort study, 101 boys and 99 girls (all aged 14 and 15 years) self-reported how often they had consumed foods containing sugar in the past month. Anthropometry, body fatness, and ASMM by dual-energy X-ray absorptiometry and metabolic risk factors, including SUA were measured. Results: Overall, 43% of girls and 57% of boys consumed ‘sugary drinks’ twice or more a day. When analyzed by group, ASMM was positively related to SUA for both boys and girls (r = 0.593, *p* < 0.0001). The effect of the intake of ‘sugary drinks’ on SUA (r = 0.176, *p* = 0.013) was reduced when ASMM was considered in the relationships. Conclusions: This study shows high SUA levels in Pacific adolescents, with a positive association between ASMM and SUA in both genders. Sugary drink intake was positively associated with SUA in both boys and girls. High ASMM in Pacific people and an increased risk for raised SUA make it important to work with Pacific communities to reduce added sugar intake and adopt integrated, family-based, culturally centered, and life-course approaches to prevent chronic diseases, including gout.

## 1. Introduction

There is growing evidence that among children and adolescents elevated serum uric acid (SUA), as the end product of purine metabolism, is a risk factor for type 2 diabetes mellitus (T2DM), hyperuricemia and gout [1,2], and cardiometabolic diseases [3]. It is also one of the factors implicated in the development of metabolic syndrome [4].

In New Zealand, the rates of hyperuricemia and gout are on the rise [5]. The prevalence of diagnosed gout is higher among adult Pacific peoples than Māori (the indigenous people of New Zealand) and European/Other New Zealand ethnic groups (Pacific = 3.9%, Māori = 3.3%, and European/Other = 1.6%) [5]. Pacific peoples living in New Zealand are a diverse-ethnic group with ancestry to the indigenous peoples of the Pacific Islands. This disproportionate burden of gout among Pacific peoples aligns with other health disparities observed in these populations, including higher rates of obesity and T2DM. The prevalence of obesity and T2DM is also increasing in these populations, with Pacific adults (>15 years) twice as likely to be obese (body mass index > 30 kg/m^2^, 67% vs. 33%) and to be diagnosed with T2DM (13.4% vs. 6.0%) than the total New Zealand population [5]. Similar to global trends [6], the age of onset of T2DM is also decreasing [7].

### 1.1. Genetic and Lifestyle Factors

Both modifiable and non-modifiable risk factors are associated with an increase in SUA. When compared with European/Other populations, genetic variants associated with decreased excretion of uric acid are prevalent in Māori and are even more prevalent in Polynesian (Māori and Pacific) populations [8,9]. In addition to genetic factors [3], body composition may also influence the relationship between modifiable factors, such as sugar-sweetened beverage intake, and serum urate levels or gout [10]. Dalbeth et al. [10] reported a high BMI (≥25 kg/m^2^) was associated with elevated SUA (*p* < 0.0001) and gout (*p* = 0.012) among individuals who frequently consumed sugar-sweetened beverages. However, these associations were not found in individuals with a low BMI. Diets high in meat, seafoods, alcohol, and fructose-containing simple sugars, may increase risk for gout, particularly in Polynesian populations [11,12,13].

The rising prevalence of obesity, metabolic syndrome, diabetes, and hyperuricemia, induced by free-sugar consumption, has been closely associated with the increased addition of free-sugars to foods over time [14]. The study by Nakagawa et al. demonstrates that uric acid plays a causal role in fructose-induced metabolic syndrome. Fructose metabolism increases uric acid production, which inhibits endothelial nitric oxide, contributing to hypertension, insulin resistance, and weight gain [15]. A daily fructose intake exceeding 50 g may contribute to metabolic syndrome and T2DM development by elevating circulating uric acid levels and promoting intracellular fat accumulation in the liver, brain, blood vessels, muscles, kidneys, and adipose tissue [16,17].

### 1.2. Adolescent Growth and SUA

Adolescence is a time of rapid growth and increases in appendicular skeletal muscle mass (ASMM) and body fat. Research has shown that Pacific peoples generally have higher appendicular skeletal muscle mass (ASMM) and lower body fat compared to other ethnic groups at the same BMI [18]. Skeletal muscle has a key role in energy metabolism [19]. Within Pacific youth, we have reported significant associations between physical function, body composition, and metabolic health during the critical period of adolescent growth and development [20]. Additionally, previous studies report that during childhood, SUA concentration increases with age, and in puberty is higher in boys than girls [3,21]. Elevated SUA concentrations during this time may be associated with lifespan incidence of T2DM [22], metabolic syndrome [3], hypertension, progression of chronic kidney disease [23], and cardiometabolic diseases [24].

Studies among adults have focused on intake of sugar-containing foods [25,26,27] and SUA but studies with children and youth are limited [1,28,29,30,31]. The primary aim of the present analysis was to investigate the associations of SUA with the consumption of sugar-containing foods and body composition among Pacific youth. We hypothesized that Pacific youth with higher SUA concentrations would have a higher intake of sugar-containing foods and higher ASMM.

## 2. Materials and Methods

The data collection approach for the Pacific Islands Families (PIFs) growth cohort study has previously been published [32]. The PIFs study tracks the growth and development of 1398 Pacific children born at Middlemore Hospital, South Auckland, New Zealand, in the year 2000. Previous measurement points have occurred at birth and ages 1, 2, 4, 6, 9 and 11 y. At age 14 y, a nested sub-study (*n* = 204, girls *n* = 100) stratified by gender and body weight decile were randomly selected from the cohort study (*n* = 931). These participants underwent assessment between May 2014 and July 2015. Selected questions quantifying sugar intake were captured from an online validated qualitative food frequency questionnaire (FFQ) that assessed food intake [33,34]. The daily consumption frequencies of sugar-containing foods were calculated from the food groups: spreads and sauces, convenience meals, biscuits/cakes, snacks and sweets, and sugary drinks (non-milk) (Supplementary Tables S1 and S2).

Fasting venous blood samples were collected and concentrations of SUA measured by the accredited Lab Plus medical laboratory (Auckland, New Zealand). SUA was measured by automated analyzer (Roche c702) using reagents, calibrators, and standard operating procedures as specified by the manufacturer. The laboratory defined the cut-off points for hyperuricemia as >0.36 mmol·L^−1^ for girls and >0.42 mmol/L for boys. Body size was measured using height, weight, and waist circumference. The BMI of each child was calculated as weight (kg) divided by the square of height (m). The Centers for Disease Control (CDC) z scores [35] were derived from BMI, age, and gender. To classify children as normal, overweight, and obese we used the cut-off values provided by Cole and Lobstein [36], as recommended by the Ministry of Health [37].

Youth underwent whole body dual-energy X-ray absorptiometry (DXA) scans for total fat, lean soft tissue, and bone mineral content (model iDXA, software version 15, GE-Lunar, Madison, WI, USA). All scans were performed and interpreted by a trained operator following standardized protocols to ensure accuracy and reliability. Total fat-free mass (FFM) was calculated from total body mass less fat mass (FM). Percent fat mass was calculated as total fat mass multiplied by 100 divided by total mass [20]. To quantify appendicular skeletal muscle mass (kg), ASMM was calculated according to Heymsfield and colleagues [38].

Data are expressed as mean and SD, unless specified otherwise. Categorical variables are reported as frequency and percentage. Independent samples T-test and Mann–Whitney U-tests were applied to determine differences by gender. The food intake distribution was normalized using log 10 (x + 1) transformation. Pearson and Spearman correlation tests were applied to assess associations. Relationships between individual food items or predefined food groups and SUA were examined visually using scatter plots and correlation analysis. Multiple linear regression (stepwise) was performed with food groups and SUA concentration controlling for gender (1 = girl, 2 = boy).

All analyses were performed using SPSS version 24 (SPSS Inc., New York, NY, USA), with statistical significance determined at a 5% level.

## 3. Results

There were no meaningful significant differences by gender in quantities of sugar- containing foods consumed, either by food groups (Table 1) or individual food items (Supplementary Table S3). Sugary drinks were consumed by the majority more than twice a day and the total frequency of sugar-containing foods consumed each day by 75% was four.

Measurements of body composition and biomarkers are shown in Table 2 with boys and girls compared. On average, boys were taller (9 cm) but not heavier than girls and had less fat mass (5 kg less), more fat-free mass (10 kg more), and more ASMM (5.5 kg more) than girls. Overall, 42% of the girls and 39% of the boys were classified as obese, with a further 34% of the girls and 32% of the boys classified as overweight. Only 24% of the girls and 29% of the boys were within the normal weight range based on the International Obesity Task Force BMI cut-offs [36].

SUA was 0.10 mmol.L^−1^higher in boys compared to girls (95%CI 0.08, 0.12; *p* = 0.001) and was higher than the gender-specific cut-off points for hyperuricemia [39] in 55% of the boys (>0.42 mmol·L^−1^) and 30% (>0.36 mmol·L^−1^) of the girls.

There were strong positive associations between SUA and all anthropometric measurements: weight z score (r = 0.453, 95% CI [0.345, 0.552], *p* = 0.001), height z score (r = 0.197, 95% CI [0.060, 0.330], *p* = 0.005) and BMI z score (r = 0.373, 95% CI [0.265, 0.480], *p* = 0.001). Adjusted for gender and age, SUA was positively related to weight (r = 0.500, 95% CI [0.395, 0.595], *p* = 0.001), height (r = 0.224, 95% CI [0.078, 0.351], *p* = 0.002), BMI (r = 0.484, 95% CI [0.381, 0.588], *p* = 0.001), waist (r = 0.502, *p* = 0.001, 95% CI [0.401, 0.597]), waist-to-height ratio (r = 0.478, 95% CI [0.376, 0.587], *p* = 0.001), FM (r = 0.476, 95% CI [0.365, 0.583], *p* = 0.001), FFM (r = 0.454, 95% CI [0.351, 0.547], *p* = 0.001), and ASMM (r = 0.469, 95% CI [0.383, 0.562], *p* = 0.001).

With the intake of sugar-containing food groups, the only meaningful correlation with SUA was for sugary drink intake (r = 0.154, 95% CI [0.016, 0.287], *p* = 0.029). Overall, 43% of girls and 57% of boys consumed ‘sugary drinks’ twice or more a day. The mean BMI z scores of these youth (*n* = 111) was 1.47 (SD = 0.937), with 26% having normal weight while 74% were classified as either overweight or obese (31% and 43%, respectively). In contrast, youth consuming sugary drinks less than twice a day (*n* = 93) had a mean BMI z score of 1.50 (SD= 0.806), 27% of whom were classified as normal weight, and 73% of whom were either overweight (35%) or obese (38%). Daily consumption of ‘sugary drinks’ was not associated with BMI (*p* = 0.813) or weight (*p* = 0.069), or by BMI cut-off categories (χ^2^ = [*df* = 370, *n* = 204], *p* = 0.403). Youth (*n* = 126) who consumed a minimum of one item from each of the five food categories (Supplementary Table S1) had a mean BMI z score of 2.00 (SD = 1.22).

**Figure 1:**
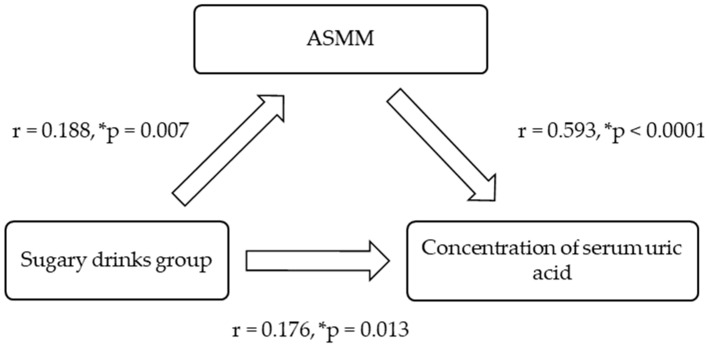
Association model for all Pacific youth (*n* = 199) controlling for appendicular skeletal muscle mass (ASMM). * Indicates statistical significance.

**Figure 2:**
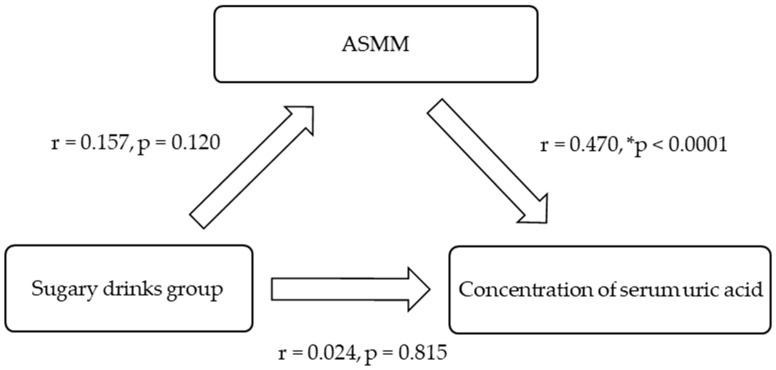
Association model for Pacific girls (*n* = 96) controlling for appendicular skeletal muscle mass (ASMM). * Indicates statistical significance.

**Figure 3:**
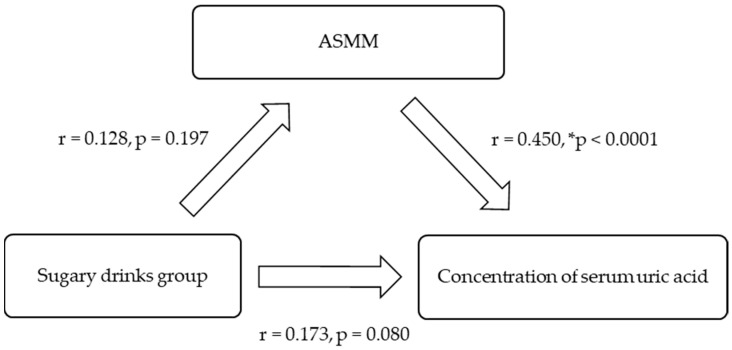
Association model for Pacific boys (*n* = 103) controlling for appendicular skeletal muscle mass (ASMM). * Indicates statistical significance.

SUA was positively and significantly related to body mass, particularly ASMM (Figure 1). The relationship between the exposure (dietary intake) and the outcome (biomarker of risk), controlled for ASMM, was explored for all youth and by gender (Figure 1, Figure 2 and Figure 3). The effect of the intake of ‘sugary drinks’ on SUA was reduced when ASMM was considered in the relationships.

The effect of consumption of food groups (Supplementary Table S1) on SUA concentration was explored using step wise regression. In unadjusted model, the ‘snacks and sweets’ and ‘sugary drinks’ food groups were negatively (standardized β −0.331, *p* = 0.001) and positively (standardized β 0.438, *p* = 0.001) related to SUA, respectively. After adjusting for gender, the following equations are obtained:

SUA concentration (mmol·L^−1^)= 0.230 + (0.092 × Gender) + (0.045 × Frequency of daily consumption of ‘sugary drinks’ group)

SEE = 0.076, adjusted R^2^ = 0.293.

Gender was coded as 1 = female, and 2 = male. Age was not a significant predictor.

## 4. Discussion

This unique study of Pacific youth aged 14 and 15 years explored the associations of sugar-containing foods and body composition with SUA concentration as part of understanding metabolic risk and physical growth. Contrary to our initial hypothesis, the strongest predictor of SUA concentration was body mass, in particular ASMM, and not sugar-containing food intake. Our analysis shows that while the ‘sugary drinks’ food group intake was initially significantly associated with SUA concentration; this effect was reduced when ASMM was included in the model. This suggests that ASMM metabolism plays a role in the relationship between sugary drinks intake, and therefore fructose consumption, and SUA. Pacific children, youth, and adults for the same BMI as other ethnic groups including European, Māori, South Asian and Chinese have less fat and more ASMM [18,20,40]. Globally, there is limited research with the unique populations of Pacific peoples at different stages of growth, so this nested cohort study, as part of a larger longitudinal study, adds to the understanding of ethnic differences in metabolism and disease risk. These findings suggest the need for more research in other non-European ethnicities to explore associations between a high ASMM, raised SUA, sugar-containing food intake, and risk for gout.

A relatively high mass of metabolically active tissues such as appendicular skeletal muscle may contribute to higher SUA levels through an increased capacity for endogenous purine turnover and storage [11]. When we analyzed this association by gender, we found no significant relationship between sugary drink intake and SUA concentration in either girls or boys. This lack of association could be influenced by gender-specific fluctuations in physiological and hormonal changes, and therefore uric acid metabolism, during puberty. At age 14 y this cohort would be well-advanced in puberty and were rapidly growing [32]. Signs of puberty among 619 members of this PIF cohort were assessed at ages 9 and 11 years using a parental questionnaire that demonstrated dramatic developmental changes [41]. Among girls, the proportion experiencing breast growth increased from 25% at age 9 to 85% at age 11. Among boys, pubertal changes such as pubic hair growth and voice deepening rose from 3% and 7% at age 9 to 16% and 18% at age 11 [41].

While other studies have shown that Polynesian peoples have a higher SUA level than other ethnic groups [8,9], this is the first time that this has been shown in Polynesian youth and for SUA to be associated with body mass and the daily consumption of substantial quantities of sugary drinks (e.g., two 330 mL cans = 70 g of sugar). As a semi-quantitative FFQ was used to assess food intake, we did not measure total food or total macronutrient intake or adjust for children with higher body mass consuming larger quantities of food and requiring higher energy intakes.

The findings in our study highlight the positive associations between sugary drink intake, SUA, and risk for gout, and ASMM measured by dual-energy X-ray, which has not been explored in other studies of adults, children, or youth. Higher ASMM is associated with a higher BMI. In a study (mean age 49.5 years), the associations between SSB intake and SUA and gout were moderated by BMI [10], with a high BMI supporting higher risk. In that study, the majority of participants were Caucasian (*n* = 10,443), with smaller representation from New Zealand Polynesian (*n* = 918) and African American (*n* = 1509) groups. Dalbeth and colleagues [10] concluded that chronic SSB intake is associated with elevated SUA and gout status in those with high BMI (≥25 kg/m^2^). Our research findings support the need for more detailed analysis of ethnicity and the specific body composition measures that contribute to these risks.

In a study of 4277 Taiwanese adolescents at ages 13–18 years from two cross-sectional Nutrition and Health Surveys in 1993–1996 and 2010–2011 the relationship between SSB consumption and SUA was examined [29]. Based on the frequency of intake of SSB (sweetened tea and soda/sports/energy drinks) participants were grouped into four groups. The authors found that, after adjusting for anthropometric measures and blood biochemistry, SUA levels were higher in adolescents who frequently consumed soda or sports drinks, regardless of whether they consumed high or low amounts of sweetened tea [29], suggesting that tea may reduce SUA. In the same analysis Shih and colleagues [29] found that mean SUA was significantly (*p* < 0.05) higher in boys compared with girls in all categories of SSB intake. However, there were no significant differences when adjusted for survey year, age, gender, physical activity (measured as metabolic equivalent per week), and total energy intake [29]. In our current study, SUA was higher in boys compared to girls (*p* = 0.001). Shih and colleagues did not address whether the mean SUA was different to the gender specific cut-off points for hyperuricemia. This may be related to the difficulties in determining a disease-specific cutoff value for uric acid levels [42].

The findings of the US National Health and Nutrition Examination Survey (US NHANES) 2001–2002 [26] with 2085 women and 1988 men aged >18 years, found that men in the highest intake quartile of estimated consumption of added sugars or SSBs had higher SUA concentrations than those in the lowest quartiles (*p* < 0.01). This association was not significant among women after adjustment for confounders such as smoking, BMI, intake of total energy, and hypertension. The authors suggested the uricosuric and hypouricemic effects of estrogen, identified in previous research, may account for this gender difference [26]. Therefore, Gao and colleagues [26] examined the association of SUA with consumption of foods with added sugar such as bread, cake, soft drinks, jam, and ice cream [43] and sugar-sweetened drinks. In men, but not women, high consumption was associated with high SUA. Foods containing natural or intrinsic sugars, such as fruits and milk, were excluded [43]. Body composition measurements were not included in this survey.

Boys had more FFM and ASMM and higher SUA concentrations compared to girls in this Pacific youth sub-study. After adjusting for age and gender, SUA concentration was found to have a significant positive association with ASMM, FFM, FM, and anthropometric measurements, including weight, height, BMI, waist circumference, and waist-to-height ratio. Krishnan and colleagues [44] conducted a fifteen-year follow-up study involving 5012 Americans aged 18–30 years at baseline to evaluate the potential of hyperuricemia as a biomarker for T2DM and impaired fasting glycemia (IFG), commonly referred to as prediabetes. At baseline (year 1986), the prevalence of obesity was 12% [44]. During the follow-up period, participants with higher SUA concentrations (>0.42 mmol·L^−1^) had higher incidence rates of T2DM (hazard ratios 1.87, 95% CI [1.33, 2.62]), insulin resistance (hazard ratios 1.36, 95% CI [1.23, 1.51]) and IFG (hazard ratios 1.25, 95% CI [1.04, 1.52]) [44]. The authors concluded that hyperuricemia in the mid-twenties may serve as an independent predictor for the development of T2DM and prediabetes. The prevalence of obesity among 14-year-olds in our Pacific youth study was higher than the prevalence in 18–30-year-olds reported by Krishnan et al. [44] (40% cf 12%). Moreover, 30% of girls and 55% of boys in the current study had SUA concentrations higher than the gender specific cut-off values. There is increasing evidence that for Pacific peoples there are population-specific gene variants associated with uric acid metabolism and gout [45] that may explain this increased risk.

A recent review of cross-sectional studies conducted among children demonstrated that higher consumption of sugary drinks was associated with a higher BMI [46]. However, in the current study, no significant relationship was found between consumption of ‘sugary drinks’ and BMI, weight, or measures of overweight and obesity, possibly because of the limitation of a selective semi-quantitative food questionnaire with no accurate measure of serving size and potential underreporting particularly by those who are obese [47]. Similarly, Shih and colleagues showed there were no significant associations between SSB intake group and anthropometric measures or blood results, including SUA [29]. However, it is important to note that their study did not measure ASMM, which could be a factor in understanding these associations.

In a nationally representative birth cohort study of Australian children, the association between the consumption of SSB or non-nutritive sweetened beverages (NNSB—artificially sweetened beverages) and subsequent overweight or obesity after two years was assessed [48]. Additionally, the study aimed to determine if the impact of SSB or NNSB consumption on overweight/obesity (from ages 2/3 to 14/15) varies between children with high and low BMI polygenic risk scores [48]. Computer-assisted dietary-recall interviews were conducted, with parents reporting for children from waves 2 to 5 (ages 2–9 years), and self-reporting beginning at wave 6 (ages 10–15 years). Children aged 4/5 to 14/15 years were classified as having obesity, overweight/obesity, or neither based on BMI z-scores using the CDC cutoff points [35].

Sycamnias et al. [48] reported that the consumption of sugar-sweetened beverages (SSBs) varied from 61% in wave 8 (aged 14–15 years) to 72% in wave 2 (aged 2–3 years). After adjusting for sex, age, socioeconomic position, breakfast consumption, adherence to guidelines for sleep duration, physical activity, polygenic risk score models (low and high), primary sample type, and the top five genetic principal components, no association was found between SSB consumption and subsequent overweight/obesity, except at age 12/13 years (*n* = 2856, *p* = 0.021, 95% CI 4.8) [48]. The authors noted that the weak association between SSB consumption and overweight/obesity might be related to the questionnaire items, which did not include all possible SSBs available to Australian children. They suggested that SSBs with higher sugar and energy content may be associated with an increased risk of obesity [48].

The strengths of our study include that the relatively short FFQ focused on commonly consumed New Zealand sugar-containing foods which reduced the time required for participants to complete the survey. Short FFQs are supported as reliable tools for measuring food intake in children and adolescents [49]. Total food consumption was not measured so total energy intake could not be evaluated or adjusted for in the regression models. In this analysis, sugary drinks included fruit juice but excluded sweetened milk beverages and other dairy products. In addition, consumption of artificial sweeteners and natural sugar substitutes were not included in the FFQ as the emphasis was on fructose and added sugar. The small sample size limited the number of statistical tests we could undertake. However, the tests undertaken were hypothesis driven.

In studies reporting association between SSB intake and SUA concentration, consumption of meat, seafood, and purine rich vegetables were also accounted for [1]. Although cross-sectional, a strength of the current study was the deliberate sampling to provide a wide range of BMI and therefore skeletal muscle mass within each gender group.

Memory based dietary assessment methods like FFQ or 24-h recalls often encounter intra-individual errors due to over- or under-reporting of specific foods but they are strongly supported food-intake assessment tools, especially in youth for assessment in the field [49]. Given the cross-sectional design of this sub-study, we are limited in our ability to track changes in sugar consumption over time and to relate this to physical growth. This design only provides a snapshot of the data at one point in time, which means that we cannot determine whether increased sugar intake causes changes in SUA levels or body composition, or whether these associations are influenced by other unmeasured factors, such as genetic predispositions or environmental influences. There is a possibility that alongside growth spurts, hormonal fluctuations, and body composition, shifts among this study population may overshadow the impacts of sugar consumption on SUA during this critical developmental phase. Therefore, future longitudinal studies, particularly within this cohort, are necessary to confirm the causality of the reported associations, to explore the observed gender-specific differences, and to investigate potential mediating factors. Such studies would provide a more comprehensive understanding of the temporal relationships and underlying mechanisms of these associations, which are crucial for informing effective public health interventions. Therefore, the strength of large, homogeneous cohort studies of participants followed from early years to later life, like the PIF study, can provide rich insight into the impact of sugar consumption on the understanding of the pathogenesis of diet-related health problems, particularly in relation to body mass and ASMM.

## 5. Conclusions

The current study adds early-in-life evidence of high SUA concentrations in Pacific youth who also have increased body mass compared with other ethnic groups. Since birth in the year 2000, the children of the PIF Study cohort have gained weight rapidly, with more than 70% classified as either overweight or obese at age 15 y [32] and many showing early signs of metabolic risk for development of non-communicable diseases. We are able to report that these youth had a high intake of sugar-containing foods, with 43% of girls and 57% of boys consuming sugary drinks twice or more per day. While findings are specific to Pacific youth living in New Zealand, they may be extended to other ethnic groups, based on migratory patterns, genetics, and ancestry [17]. However, further research is needed to examine whether these associations hold across similar age groups in other ethnic populations with different genetic and cultural backgrounds.

In light of these findings, reducing sugar intake is crucial [14,50] as it represents a key component of a poor-quality diet. Adolescence and youth is a critical period for establishing lifelong dietary habits. The negative impacts of a poor-quality diet, such as those high in sugar, on metabolic health might not be immediately apparent, with some analyses indicating that cardiometabolic risk factors may only emerge in late adolescence or later in life [51]. Public health guidance and consumer communication have suggested focusing on ‘free-sugars’ as a key component of dietary recommendations [52]. These support the need for robust public health interventions focused on reducing sugar intake among Pacific youth.

Integrated, family-based, culturally centered and life-course approaches with emphasis on community involvement, such as the Pasifika Prediabetes Youth Empowerment Program (PPYEP) [53], are essential for addressing the health and social needs of Pacific families and thereby improving long-term health outcomes in vulnerable populations.

## Figures and Tables

**Table 1:** Daily reported consumption of sugar-containing food groups by gender.

Predefined Free-Sugar Food Groups	Total (n = 204)	Girls (n = 100)	Boys (n = 104)	*p* Value ^b^
	Median (IQR) ^a^	Median (IQR)	Median (IQR)	
Spreads and sauces	1.07 (0.64, 2.05)	1.00(0.63, 1.75)	1.08 (0.63, 2.40)	0.327
Convenience meals	0.28 (0.08, 0.79)	0.22 (0.07, 0.64)	0.43 (0.07, 0.98)	0.207
Biscuits/cakes	1.38 (0.84, 2.52)	1.35 (0.92, 2.22)	1.43 (0.64, 2.94)	0.889
Snacks and sweets	0.57 (0.26, 1.28)	0.67 (0.36, 1.20)	0.49 (0.23, 1.41)	0.093
Sugary drinks	2.12 (1.13, 3.64)	1.89 (0.99, 3.03)	2.35 (1.44, 4.22)	0.207
Total sugar-containing foods	5.92 (3.81, 10.19)	5.73 (3.87, 8.98)	6.09 (3.67, 11.07)	0.889

^a^ IQR; interquartile range, ^b^ Mann–Whitney U test.

**Table 2:** Characteristics of Pacific youth (n = 204).

	Girls (n = 100)		Boys (n = 104)		*p* Value ^d^
	Mean	SD	Mean	SD	
**Age (years)**	14.92	0.47	14.88	0.43	0.481 ^e^
**Anthropometry**					
Weight (kg)	81.2	20.6	85.8	25.2	0.246
Height (cm)	166.6	5.6	175.4	7.1	**0.001** ^e^
BMI (kg·m^−2^)	29.1	6.5	27.7	7.5	0.073
Waist (cm)	84.5	16.2	88.6	18.8	0.191
Waist/height (cm)	0.50	0.09	0.50	0.10	0.570
Weight z scores ^a^	1.68	0.75	1.79	1.18	0.443 ^e^
Height z scores ^a^	0.75	0.86	0.81	0.90	0.576 ^e^
BMI z scores ^a^	1.55	0.70	1.42	1.02	0.263 ^e^
**Body composition** ^b^					
Fat free mass (kg)	49.7	8.4	60.0	11.3	**0.001** ^e^
Fat mass (kg)	31.3	13.0	25.9	15.6	**0.001**
Fat mass%	37.3	6.5	28.0	9.3	**0.001** ^e^
Appendicular skeletal muscle mass (kg)	21.1	4.5	26.6	5.8	**0.001** ^e^
**Biomarker**					
SUA (mmol·L^−1^) ^c^	0.33	0.63	0.43	0.09	**0.001** ^e^

^a^ CDC growth charts [35]. ^b^ Measured by dual-energy X-ray absorptiometry. ^c^ SUA; serum uric acid. SUA was not available for four girls and one boy. ^d^ Mann–Whitney U test. ^e^ Independent T-test. Significant *p*-values (*p* < 0.05) are bolded in the table.

## Data Availability

The datasets presented in this article are not readily available. Requests to access the datasets should be directed to the Co-directors of the Pacific Islands Families study.

## Supplementary Materials

The following supporting information can be downloaded at: https://www.mdpi.com/article/10.3390/nu17010054/s1, Table S1. Predefined sugar-containing food groups based on 38 foods in the frequency questionnaire. Table S2. Frequency of consumption of food and the applied weighting factor to standardize to a daily rate. Table S3. Daily frequency of consumed foods by gender.
