# Supplementary material for: Pacific Islands Families Study: Serum Uric Acid in Pacific Youth and the Associations with Free-Sugar Intake and Appendicular Skeletal Muscle Mass
Source: Nutrients. 2024 Dec 27;17(1):54. doi: 10.3390/nu17010054 (PMC11722811; doi:10.3390/nu17010054)
Supplement: Supplementary file 1 [file nutrients-17-00054-s001.zip › nutrients-3361846-supplementary.pdf]

Supplementary materials

Supplementary Table S1

Predefined sugar-containing food groups based on 38 foods in the frequency questionnaire

Predefined sugary food groups	Food items
Spreads and sauces	jam or honey, Nutella™, Marmite or Vegemite, peanut butter, mayonnaise or salad dressing, tomato sauce or ketchup and other spreads or sauces
Convenience meals	canned spaghetti with tomato sauce, baked beans and 'other' convenience meals
Biscuits/ cakes	chocolate coated or cream filled biscuits, biscuits e.g., plain, bars e.g. muesli bar, crackers or crispbreads, cake or slice, doughnuts or croissants, scones, muffins or sweet buns, pancake or pikelets, fruit pie, fruit crumble or tart, pudding, custard or custard puddings and other item of the biscuits/ cakes
Snacks and sweets	popcorn, chocolate, candy coated chocolate and 'other sweets'
Sugary drinks [a]	juice, powdered fruit drink, fruit drink from concentrate or cordial, Coca Cola or other cola drinks, Mountain Dew, 'New Age' drinks, soft drinks, sports drinks, ice blocks, tea, coffee and 'other drinks'

[a] Contain free sugar. It is named 'sugary drinks' in the current study excluding sweetened milk drinks and dairy products.

Supplementary Table S2

Frequency of consumption of food and the applied weighting factor to standardise to a daily rate.

Frequency t	Weight
Never or less than once a month	0.005
1 to 3 times a month	0.067
1 to 2 times a week	0.214
3 to 4 times a week	0.5
5 to 6 times a week	0.786
Once a day	1
2 or more times a day	2

Supplementary Table S3

Daily frequency of consumed foods by gender.

	Daily frequency [a] Median (percentile 25th, 75th)			
	**Total n = 204**	**Girls n = 100**	**Boys n =104**	***p* Value** [b]
**Spreads and sauces**				
Jam or honey	0.07 (0.01, 0.21)	0.07 (0.01, 0.21)	0.07 (0.01, 0.21)	0.871
Nutella	0.01 (0.01, 0.01)	0.07 (0.01, 0.21)	0.07 (0.01, 0.50)	0.083
Marmite or Vegemite	0.07 (0.01, 0.21)	0.01 (0.01, 0.01)	0.01 (0.01, 0.05)	0.382
Peanut butter	0.07 (0.07, 0.21)	0.01 (0.01, 0.21)	0.07 (0.01, 0.21)	**0.040**
Mayonnaise or salad dressing	0.21 (0.07, 0.50)	0.21 (0.07, 0.21)	0.07 (0.07, 0.21)	0.531
Tomato sauce or ketchup	0.00 (0.00, 0.00)	0.50 (0.21, 0.50)	0.21 (0.07, 0.79)	0.528
Other spreads or sauces	0.07 (0.01, 0.21)	0.00 (0.00, 0.00)	0.00 (0.00, 0.00)	0.959
Total	1.07 (0.64, 2.05)	1.00 (0.63, 1.75)	1.08 (0.63, 2.40)	0.392
**Convenience meals/snacks**				
Canned spaghetti with tomato sauce	0.07 (0.01, 0.21)	0.07 (0.01, 0.21)	0.07 (0.01, 0.21)	0.889
Baked beans	0.01 (0.01, 0.07)	0.01 (0.01, 0.07)	0.01 (0.01, 0.07)	0.939
Other Convenience meals/snacks	0.07 (0.00, 0.50)	0.07 (0.00, 0.50)	0.21 (0.00, 0.50)	0.129
Total	0.28 (0.08, 0.79)	0.22 (0.07, 0.64)	0.43 (0.07, 0.98)	0.271
**Biscuits/ cakes**				
Chocolate coated or cream filled biscuits	0.21 (0.07, 0.50)	0.21 (0.07, 0.5)	0.21 (0.07, 0.50)	0.694
Biscuits e.g. plain	0.21 (0.07, 0.50)	0.21 (0.07, 0.5)	0.07 (0.07, 0.50)	0.343
Bars e.g. muesli	0.21 (0.01, 0.50)	0.07 (0.01, 0.5)	0.21 (0.07, 0.50)	0.212
Crackers or crispbreads	0.21 (0.07, 0.50)	0.21 (0.07, 0.5)	0.21 (0.02, 0.50)	0.546
Cake or slice	0.07 (0.07, 0.21)	0.07 (0.07, 0.21)	0.07 (0.01, 0.21)	0.566
Doughnuts or croissants	0.07 (0.01, 0.07)	0.07 (0.01, 0.07)	0.07 (0.01, 0.07)	0.859
Scones, muffins or sweet buns	0.07 (0.01, 0.21)	0.07 (0.01, 0.21)	0.07 (0.01, 0.07)	**0.027**
Pancake or pikelets	0.07 (0.01, 0.21)	0.07 (0.01, 0.21)	0.07 (0.01, 0.21)	0.524
Fruit pie, fruit crumble or tart	0.01 (0.01, 0.01)	0.01 (0.01, 0.01)	0.01 (0.01, 0.07)	0.071
Pudding	0.01 (0.01, 0.07)	0.01 (0.01, 0.07)	0.01 (0.01, 0.07)	0.491
Custard or custard puddings	0.01 (0.01, 0.07)	0.01 (0.01, 0.07)	0.01 (0.01, 0.07)	0.632
Other item of the biscuits/ cakes	0.00 (0.00, 0.00)	0.00 (0.00, 0.00)	0.00 (0.00, 0.00)	0.429
Total	1.38 (0.84, 2.52)	1.35 (0.92, 2.22)	1.43 (0.64, 2.94)	0.987
**Snacks and sweets**				
Popcorn	0.07 (0.01, 0.21)	0.07 (0.01, 0.21)	0.07 (0.01, 0.21)	0.342

	Daily frequency [a] Median (percentile 25th, 75th)			
	**Total** **n = 204**	**Girls** **n = 100**	**Boys** **n =104**	***p* Value** [b]
Chocolate	0.21 (0.07, 0.50)	0.21 (0.07, 0.50)	0.21 (0.07, 0.50)	0.340
Candy coated chocolate	0.07 (0.07, 0.21)	0.14 (0.07, 0.21)	0.07 (0.01, 0.21)	0.113
Other sweets	0.21 (0.07, 0.50)	0.21 (0.07, 0.50)	0.07 (0.01, 0.21)	**0.012**
Total	0.57 (0.26, 1.28)	0.67 (0.36, 1.20)	0.49 (0.23, 1.41)	0.065
**Sugary drinks**				
Juice	0.21 (0.07, 0.50)	0.14 (0.07, 0.50)	0.21 (0.07, 0.50)	0.058
Powdered fruit drink	0.21 (0.07, 0.50)	0.21 (0.07, 0.50)	0.21 (0.07, 0.50)	0.346
Fruit drink from concentrate or cordial	0.07 (0.01, 0.21)	0.07 (0.01, 0.21)	0.07 (0.01, 0.50)	0.266
Coca cola or other cola drinks	0.21 (0.07, 0.50)	0.07 (0.01, 0.50)	0.21 (0.07, 0.50)	0.099
Mountain Dew	0.07 (0.01, 0.50)	0.07 (0.01, 0.21)	0.21 (0.07, 0.50)	0.066
'New Age' drinks	0.01 (0.01, 0.07)	0.01 (0.01, 0.01)	0.01 (0.01, 0.07)	**0.048**
Soft drinks	0.21 (0.07, 0.50)	0.21 (0.07, 0.50)	0.21 (0.07, 0.50)	0.344
Sports drinks	0.07 (0.01, 0.21)	0.07 (0.01, 0.21)	0.07 (0.07, 0.21)	**0.015**
Ice blocks	0.07 (0.01, 0.21)	0.07 (0.01, 0.21)	0.07 (0.01, 0.21)	0.275
Tea	0.07 (0.01, 0.50)	0.07 (0.01, 0.50)	0.07 (0.01, 0.50)	0.882
Coffee	0.01 (0.01, 0.07)	0.01 (0.01, 0.18)	0.01 (0.01, 0.07)	0.186
Other item of the 'Other drinks'	0.00 (0.00, 0.07)	0.00 (0.00, 0.07)	0.00(0.00, 0.01)	0.746
Total	2.12 (1.13, 3.64)	1.89 (0.99, 3.03)	2.35 (1.44, 4.22)	**0.034**

[a] Frequency of foods consumed was assumed as frequency of consumption of a serving of that food

[b] Mann-Whitney U test.
